# A novel remote sensing-based approach to determine loss of agricultural soils due to soil sealing — a case study in Germany

**DOI:** 10.1007/s10661-024-12640-z

**Published:** 2024-05-04

**Authors:** Annelie Säurich, Markus Möller, Heike Gerighausen

**Affiliations:** Institute for Crop and Soil Science, Julius Kühn Institute (JKI) – Federal Research Centre for Cultivated Plants, Bundesallee 58, Braunschweig, Lower Saxony 38116 Germany

**Keywords:** Soil loss, Sustainable land use, Soil functions, Soil evaluation, High-Resolution Imperviousness layers, Copernicus Land Monitoring Service

## Abstract

Soils provide habitat, regulation and utilization functions. Therefore, Germany aims to reduce soil sealing to 30 ha day^-1^ by 2030 and to eliminate it by 2050. About 55 ha day^-1^ of soil are damaged (average 2018–2021), but detailed information on its soil quality is lacking. This study proposes a new approach using geo-information and remote sensing data to assess agricultural soil loss in Lower Saxony and Brandenburg. Soil quality is assessed based on erosion resistance, runoff regulation, filter functions, yield potential and the Müncheberg Soil Quality Rating from 2006 to 2015. Data from the German Soil Map at a scale of 1:200,000 (BÜK 200), climate, topography, CORINE Land Cover (CLC) and Imperviousness Layer (IMCC), both provided by the Copernicus Land Monitoring Service (CLMS), are used to generate information on soil functions, potentials and agricultural soil loss due to sealing. For the first time, soil losses under arable land are assessed spatially, quantitatively and qualitatively. An estimate of the qualitative loss of agricultural soil in Germany between 2006 and 2015 is obtained by intersecting the soil evaluation results with the quantitative soil loss according to IMCC. Between 2006 and 2015, about 73,300 ha of land were sealed in Germany, affecting about 37,000 ha of agricultural soils. This corresponds to a sealing rate of 11 ha per day for Germany. In Lower Saxony and Brandenburg, agricultural soils were sealed at a rate of 1.9 ha day^-1^ and 0.8 ha day^-1^ respectively, removing these soils from primary land use. In Lower Saxony, 75% of soils with moderate or better biotic yield potential have been removed from primary land use, while in Brandenburg this figure is as high as 88%. Implementing this approach can help decision-makers reassess sealed land and support Germany’s sustainable development strategy.

## Introduction

Although soils are only a thin layer on the Earth’s surface, they are dynamic systems of great importance to all terrestrial life, including humans, and provide a wide range of services (Blum, 2005; Hatfield et al., 2017). Soils are most worthy of protection, especially since soils are a non-renewable resource in a human timescale (Food and Agriculture Organization of the United Nations, 2015). Regardless of this, soils around the world are subject to degradation processes caused by human activity, which irreversibly alters or, in the worst case, destroys them (Bridges & Oldeman, 1999).

Because soil degradation is a slow and gradual phenomenon, it does not receive as much attention as natural hazards such as floods, earthquakes or tsunamis, even though the consequences are much more severe (Keesstra et al., 2016). The importance of soil science to ensure planetary and human well-being is visible in the global issues addressed by the United Nations Sustainable Development Goals (SDGs, United Nations General Assembly, 2015) as half of the targets relate to soil. The European Union (EU) Soil Strategy for 2030 outlines the long-term vision to ensure that all soils are healthy by 2050, and to make protecting, using and restoring soils the norm, while also pursuing the SDG 15.3 targets (European Commision, 2021). The SDG 15.3 focuses on the concept of “Land Degradation Neutrality”, namely to “combat desertification, restore degraded land and soil, including land affected by desertification, drought and floods, and strive to achieve a land degradation-neutral world” (United Nations General Assembly, 2015). However, measures to halt and reverse land degradation (SDG 15.3) are usually expensive and do not lead to immediate improvements. In addition, assessing and monitoring soil quality can be challenging due to the heterogeneous nature of soils. Good monitoring methods that are reliable, standardized, meaningful, robust, comprehensible and repeatable can be expensive and time consuming (Keesstra et al., 2016). For these reasons, a directive on Soil Monitoring and Resilience was proposed in the EU in July 2023 as part of the EU Soil Strategy, suggesting a robust and coherent soil monitoring framework across the EU (European Commision, 2023). As the EU risks failing to meet its international and European commitments on the environment, sustainable development and climate change if the soil is not properly protected, the proposed Soil Monitoring Law aims to fill the current knowledge gaps and provide the essential data and information needed to take the right actions to achieve the SDGs and the EU Soil Strategy 2030. In Germany, these commitments are implemented in the National Sustainable Development Strategy of Germany (Nachhaltigkeitsstrategie, Bundesregierung, 2016). This agenda aims, among other things, to protect and sustainably use soil as a resource and seeks to reduce land use for settlement and transport. The sustainable preservation of soil fertility and soil performance as a natural resource is further regulated in the Federal Soil Protection Act (BBodSchG, 1998) and is known as Good Agricultural Practice.

Soil sealing is one of the main processes of the 17 pathways of soil degradation (Prăvălie, 2021). There are a lot of different definitions and wordings for the phenomenon including impervious surface, soil sealing, land take, soil consumption and land consumption which are further described in Peroni et al. (2022). They all imply loss of soil in the sense that the land can no longer be used for its original purpose for the time being or, in the worst case, can no longer be used for its original purpose at all. In Germany, soil sealing is the preferred term, which describes land that is built over or paved (e.g., water-bound surfaces, asphalted, concreted or paved surfaces) (Frie & Hensel, 2009). In the present study, the term soil sealing is used as a description and synonym for the loss of soil due to built-up areas in relation to the original agricultural use of the land, following the definition of Frie and Hensel (2009) and Peroni et al. (2022). According to this definition, soil loss through erosion is not taken into account in this study. In contrast to natural soil sealing, where the water infiltration in the soil is hindered due to soil compaction, dispersion of colloids or rain impact, artificial soil sealing is extensive and permanent (Scalenghe & Marsan, 2009). Artificial soil sealing majorly affects the proper functioning of the soil, leaving the soil and ecosystem functions immensely damaged or even nullified in the worst case. Moreover, these sealed soils are disconnected from other neighboring environmental compartments leading to negative effect including loss of water retention areas, less carbon sequestration, loss of biodiversity, less available fertile soils for future generations, and many more (Prokop et al., 2011). The increasing importance and extent of landscape conversion in Europe is particularly evident in the Netherlands, Belgium and Germany where there are exceptionally high losses of agricultural soils due to sealing (European Environment Agency, 2002; European Union, 2012).

Quantifying soil loss by sealing is possible using one of the three general approaches (Behnisch et al., 2016; Peroni et al., 2022): (i) high-resolution remote sensing data in combination, for example, with vegetation index analysis, spectral mixture analysis or regression trees (Franke et al., 2009; García & Pérez, 2016), (ii) a multi-criteria approach combining remote sensing data of moderate resolution and accurate sealed surface maps for a representative part of the desired area (Hartcher & Chowdhury, 2017; Krówczyńska et al., 2016; Nickayin et al., 2021), or (iii) a pre-determined indicator to calculate soil sealing based on the area survey according to the type of actual use (Müller et al., 2010; Pristeri et al., 2020; Gerundo & Grimaldi, 2011). The European Earth observation program Copernicus provides manifold resources for different fields of application for satellite-based data products and services which can be used for the high-resolution remote sensing approach (Apicella et al., 2022). With CORINE Land Cover (CLC), the Copernicus Land Monitoring Services (CLMS) provides pan-european information on land cover (European Union, 2023a). Further, the High-Resolution Layers (HRL) of CLMS provide information from satellite imagery about different land cover characteristics that is sealed (impervious) surfaces. In support of sustainable agriculture and climate change mitigation, Copernicus offers the potential for additional information and data products on land cover/land use classification (European Environment Agency, 2021a; European Union, 2023a; Federal Agency for Cartography and Geodesy (BKG), 2018), crop type classification (Asam et al., 2022; Blickensdörfer et al., 2022; d’Andrimont et al., 2021; Preidl et al., 2020), derivation of soil information (Castaldi et al., 2019; Dvorakova et al., 2023; Safanelli et al., 2020; Zepp et al., 2021) or soil moisture properties (Nativel et al., 2022; Pulvirenti et al., 2018).

A sustainability indicator for target 11.1.a in the German National Strategy for Sustainable Development “increase in settlement and transport area” (Bundesregierung, 2016; Federal Statistical Office (Destatis), 2022b) is used to monitor the goal of Germany to reduce land use for settlement and transport to an average of 30 ha per day until 2030 and to achieve a circular economy in 2050. According to this indicator, Germany had an average loss of 55 ha of valuable soils per day between 2018 and 2021 (Federal and State Statistical Offices, 2023a). Although the total amount of soil sealing in Germany is known within the framework of the sustainability indicator (Federal Statistical Office (Destatis), 2022a), there is a lack of information on the exact location and previous soil quality of the sealed soils, as there is currently no local-scale and nationwide information on soil loss.

Soil functions and potentials are used as indicators of soil quality in this study according to Karlen et al. (1997), who defined soil quality as “the capacity of a specific kind of soil to function, within natural or managed ecosystem boundaries, to sustain plant and animal productivity, maintain or enhance water and air quality, and support human health and habitation”. Monitoring changes in soil properties over time due to management for different soils and land uses is an important aspect of soil quality evaluation (Andrews et al., 2004). Although there are numerous previous assessments of soil quality in the literature, the majority of studies focus on a single plot or site (e.g., Askari and Holden, 2014; Lima et al., 2013; Rutgers et al. ; Shukla et al., 2012; 2006) and in most cases extrinsic factors, such as site-specific characteristics, management, or climatic data are ignored (e.g., Armenise et al., 2013; Cotching and Kidd, 2010; Juhos et al., 2019; Santos-Francés et al., 2019).

In the present study, we aim to fill this knowledge gap by assessing the soil functions and potentials of agricultural soils in Germany, using the best possible resolution of a consistent soil map for the whole of Germany. To provide national and regional information on yield capacity (biotic yield potential and the Müncheberg Soil Quality Rating) and soil vulnerability to (anthropogenic) changes in the landscapes budget (erosion resistance, runoff regulation, filter functions), and to quantify the “cost” of soil loss in terms of soil quality, we are exploring various existing and newly available digital data sources and the adaptation and enhancement of existing soil functions and potentials. For the first time, soil loss due to sealing for building and infrastructure development will be assessed spatially, quantitatively and qualitatively by combining different geospatial and remote sensing data. This in turn can then support the targets of the EU Soil Strategy and the related Soil Monitoring Framework, e.g., indicators on soil sealing and reduced land use.

**Fig. 1:**
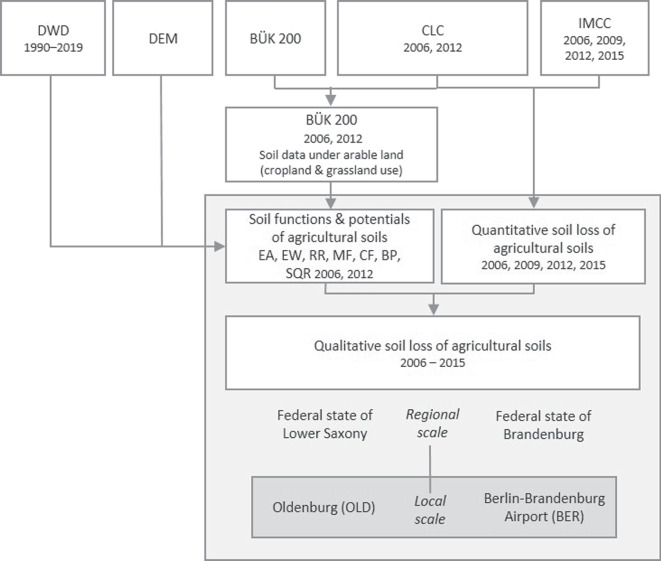
Schematic work flow to determine the quantitative and qualitative soil losses of agricultural soils in Lower Saxony and Brandenburg and local scale test areas of Oldenburg (OLD) and Berlin-Airport (BER) in these two federal states. As stated underneath each of the input data, they are available for different years. EA: erosion resistance against water, EW: erosion resistance against wind, RR: runoff regulation function, MF: mechanical filter function, CF: physical-chemical filter function, BP: biotic yield potential, SQR: Müncheberg Soil Quality Rating, CLC: CORINE Land Cover, BÜK 200: soil map of Germany (“Bodenübersichtskarte”) 1:200,000, DWD: Germany’s National Meteorological Service, DEM: Digital Elevation Model, IMCC: Imperviousness Classified Change

## Materials and methods

### General approach

In the present study, a comprehensive analysis and processing of different data sources is carried out to evaluate different soil functions and potentials in order to subsequently make statements about the quantitative and qualitative loss of agricultural soils (Fig. 1). The holistic approach is presented on a regional scale as an example for the federal states of Lower Saxony and Brandenburg (4,761,400 ha and 2,947,900 ha, respectively). For a more detailed investigation and evaluation of soil losses on a local level, two local test areas with different sealing behaviors were selected, each comprising of two $10\times 10$ km grid cells (each site 20,000 ha). One area is in the vicinity of Oldenburg in Lower Saxony (OLD) and has a combination of rural and urban sealing and a scattered structure. The other area includes the recently completed Berlin-Brandenburg Airport in Brandenburg (BER) and thus contains a large amount of contiguous sealed land used primarily for infrastructure. However, the approach can in principle be applied to the entire area of Germany.

Firstly, the soil functions and potentials (see “Soilfunctions and potentials” section, Marks et al. 1992; Müller et al. 2007) are determined for soils under arable land. Land use data (CORINE Land Cover, CLC, European Union 2023a, see “Copernicus land monitoring service” section), soil information (soil map of Germany (“Bodenübersichtskarte”) on the scale of 1:200,000, BÜK 200, Federal Institute for Geosciences and Natural Resources (BGR) 2021, see “Soil, climateand terrain data” section), climate data (Germany’s National Meteorological Service, DWD, see “Soil, climate and terrain data” section) and topographic data (Digital Elevation Model, DEM, Federal Agency for Cartography and Geodesy (BKG) 2016, see “Soil, climate and terrain data” section) are used as input variables in the process. Secondly, the quantitative soil loss is assessed with the Imperviousness Classified Change Layers (IMCC) of the Copernicus Land Monitoring Service (CLMS, European Union 2023a) and the CLC data. The intersection of the soil evaluation results and the quantitative soil loss according to IMCC gives an estimation of the qualitative loss of agricultural soil in Germany between 2006 and 2015. Additionally, non-satellite-based data of the German Main Land-use Survey from the Federal Statistical Office are used as reference data on soil loss quantity in Germany to estimate the accuracy of the IMCC data (see “Federal statisticaloffice data” section).

### Soil functions and potentials

The present study uses the soil evaluation approaches of Marks et al. (1992) and Müller et al. (2007). Both combine the ability to consider both intrinsic (soil properties) and extrinsic (climate, land use, site-specific data) factors and soil threats. Incorporating extrinsic factors and including measures of ecosystem services and soil threats is important for a comprehensive soil quality assessment (Bünemann et al., 2018). Furthermore, the two used approaches are conceptualized for soil assessments on a large scale and can be implemented for various soils and regions in Germany. Another major advantage of them is their comprehensive and consistent handling of input data (see Fig. 9 in the Appendix). This is the first time that the approaches have been used with the BÜK 200 soil data.

A total of seven different soil functions and potentials were picked for the evaluation. The biotic yield potential (Marks et al., 1992) and the Müncheberg SQR (Müller et al., 2007) are used to estimate the yield capacity. Furthermore, five soil functions from Marks et al. (1992) are applied to evaluate the vulnerability of soil due to intrinsic and extrinsic factors. The evaluation is carried out according to a ranking scale with the evaluation scores 1–5 (1–6 for erosion resistance against water or wind) representing very low to very high capacity, respectively. All information and calculations in “Erosion resistance to Bioticyield potential” sections are taken from Marks et al. (1992) unless otherwise noted.

#### Erosion resistance

Erosion resistance describes the capacity or ability to counteract soil erosion caused by water or wind beyond the natural measure. Due to agricultural use and temporarily less coverage of soil area with vegetation and therefore less protection, soil erosion exceeds its natural state. The manner in which soil properties offer resistance depends on the type of erosion (water or wind erosion).

##### Erosion resistance against water

The extent of soil erosion by surface runoff of precipitation and melt-water is determined by the erosion potential of the precipitation and the erodibility of the site in question. Erosion resistance is essentially based on the effect of the soil and topographic factor, plus the effects of management. The foundation of this indicator is formed by the universal soil loss equation (USLE, Wischmeier and Smith, 1978), however the here used calculation of the mean annual soil loss *A* is based on a simplified version of the USLE according to Eq. 1

1 $$\begin{aligned} A = K \times LS \times R \times C. \end{aligned}$$

In addition to the main erosion factors that are closely related to soil type and soil texture, namely aggregate size and stability, infiltration and permeability, humus and coarse soil content (proportion of particles with a size > 2 mm) also affect erosion resistance. These soil properties represent the soil erodibility factor *K* and are used to calculate the erosion resistance due to soil type-related values. The erosion resistance resulting from the topography is primarily a consequence of the slope inclination and surface shape. Therefore, the slope-length and slope-steepness factor *LS* takes curvature (convex, concave), slope inclination and an average slope length of 100 m into account when the mean soil loss values are calculated. Further, these values are corrected by the rainfall and runoff factor *R*, which is based on the summer precipitation, and by the cover and management factor *C* depending on the land use and the overall soil depth.

##### Erosion resistance against wind

Soil erosion by wind occurs mainly on sandy soils with particle sizes < 1  mm and on degraded peatlands under agriculture. Increased silt and clay content, elevated humus content and increasing water content of the upper soil layer can increase erosion resistance by strengthening both cohesive forces and colloid content of the soil. Erosion resistance of mineral soils is calculated using soil type, humus content and ecological moisture content. For peatlands under agricultural use, vulnerability increases with increasing degree of decomposition caused by degradation. Furthermore, fen peat is assumed to be more susceptible to wind than bog peat.

#### Runoff regulation

The runoff regulation function implies the ability to retain surface water in the ecosystems by reducing the immediate runoff and thereby contributing to balanced runoff conditions. These are characterized by both climatic conditions and area properties. The amount and intensity of precipitation events, as well as the location of the area in relation to the direction of movement of the precipitation field, are critical factors in peak runoff. Further impact factors are soil coverage, slope, soil moisture, and infiltration capacity of the area. Meteorological data, soil coverage and soil moisture status are short-term and time-variable factors that can therefore not be included in these calculations. However, land use, slope inclination, soil type and effective field capacity are used to evaluate runoff.

#### Filter, buffer and transformation function

These functions include the ability of the soil to protect the subsoil from the penetration of undesirable substances, due to the low permeability of the soil or the degradation of these substances, or due to a good buffering or filtering capacity. Suspended soil and pollutant particles can be mechanically bound in the soil, whereby the soil can be used as surface and/or heap filter. The buffer function of the soil refers to the ability to bind pollutants or surplus nutrients in dissolved or gaseous form by adsorption to the soil exchangers. Further, the buffer function includes chemical precipitation of this matter after reaction with the soil’s own substances and thus its extensive immobilization.

##### Mechanical filter function

The mechanical filter function describes the suitability of the soil for mechanical clarification of a suspension. It is assessed on the basis of its water permeability and the proportion of self-draining pores. These physical characteristics are ascribed to soil or peat type. Each soil layer down to the groundwater table is evaluated individually. Subsequently the weighted average is calculated. In addition, the filter distance above the groundwater table and the climatic water balance surplus are taken into account.

##### Physical-chemical filter function

The physical-chemical filter effect is the ability of soil to absorb dissolved substances from the soil solution. The absorption capacity depends primarily on the surface activity of the soil particles. The basis of the estimation is therefore the sorption capacity of the soil, which can be approximated largely by using the cation exchange capacity of the soil type. In addition, the filter distance above the groundwater level is used. Analogous to the mechanical filter function each soil horizons down to the groundwater table is evaluated individually. Subsequently, the weighted average is calculated.

#### Biotic yield potential

The biotic yield potential describes the capacity of the landscape to produce biomass that can be utilized and to ensure the ongoing repeatability of this process for sustainability. The assessment is based on the state of the site factors that influence the type of land use, yield and production costs. Furthermore, the possibilities of the site being endangered by soil erosion are included. Factors of the topography (soil inclination), the soil (coarse soil content, soil depth, soil type), variables influencing the water regime (depth of water table, waterlogging, effective field capacity) and the climate (mean temperature, annual precipitation) are taken into account. An average intensity of cultivation is assumed. The factors are aggregated to an overall value, whereby the least favorable factor is always decisive.

#### Müncheberg soil quality rating (SQR)

Additionally to the biotic yield potential the Müncheberg SQR (Müller et al., 2007) was determined. This assessment was chosen since it is a well-known, proven and tested soil quality tool with lots of input variables. However, it is focused mainly on cropping land use under wheat while grassland is given less priority scoring-wise. Furthermore all input variables must be present otherwise the scoring is not possible. To nevertheless obtain comprehensive nationwide soil potentials we calculated both, the biotic yield potential and the SQR while simultaneously considering several different input variables.

The Müncheberg SQR is a simple method to rate soil quality of farmland. It was developed to estimate the suitability of soils for agricultural use and to roughly estimate the potential yield using only a given pedon in the field. However, the pedon rating can be transferred to landscapes. Based on a set of variables and sub-indicators, which take into account soil profile features, as well as hydrology and topography of the area around the soil pedon, the final SQR score evaluates the long-term soil quality and is given within a 100 point scale. In a first step, the SQR basic score is calculated by weighted sum of eight basic soil variables (Fig. 9 in the Appendix). Soil properties and layering are the main components of the basic soil variables along with topography and water budget information. The empirical additive approach is maintainable because most German soils are prime arable land, and therefore basic soil parameters only vary in certain ranges and soil quality will not be inhibited by an individual basic variable. In a second step, hazardous soil properties and factors, which may limit the soil quality, are considered. Often these hazard factors are linked to climatic variables. However, for most soils in Germany soil quality is not impaired by these factors. Here, we focused on a subset of four hazard factors: acidification, soil depth above hard rock, drought risk and coarse soil texture fragments, respectively, as suggested by Richter et al. (2009). In the overall SQR score, the hazard factors are integrated as multipliers. The lowest multiplier is the applied factor that is multiplied with the basic soil score. To facilitate comparisons with the soil functions and potential the 100 point scale is translated into 5 classes (0–20, 20–40, 40–60, 60–80, 80–100) as suggested by Müller et al. (2007).

### Data collection and data processing

#### Soil, climate and terrain data

Required comprehensive soil information were obtained from the freely available nationwide soil map of Germany (“Bodenübersichtskarte”) on the scale of 1:200,000 (BÜK 200, Federal Institute for Geosciences and Natural Resources (BGR), 2021). To date this map is the highest resolution consistent soil map of Germany. In the BÜK 200 database, a number of similar soil profiles are grouped under one universal key, which is connected to the shape file. The soil profiles and associated soil properties of each soil layer can be assigned by using the land use mapped in the CORINE Land Cover (CLC, European Union, 2023a) inventory of 2006 and 2012 (see “Copernicus land monitoringservice” section). Therefore, the land use of the CLC and BÜK 200 were intersected for grassland and cropland (see “Copernicus land monitoring service” section for details). Only polygons matching these six CLC classes were considered in the attribution. Depending whether the mapped land use in the BÜK 200 and CLC is concise or not, the soil profiles are assigned according to a standardized workflow. Given that the BÜK 200 is lacking a considerable amount of water table information, the respective missing values were gathered from the nation wide soil map on the scale of 1:1,000,000 (BÜK 1000, Federal Institute for Geosciences and Natural Resources (BGR), 2007), alternatively.

All relevant climatic data originate from Germany’s National Meteorological Service (DWD) in a resolution of 1 km $\times$ 1 km. This concerns the variables “annual sum of precipitation”, “mean annual temperature”, “summer precipitation” (May-October), “potential evaporation” and “climatic water balance” (difference between precipitation and potential evaporation). The present study was conducted using long-time averages from 1990–2019 for all climatic variables.

The terrain attributes “slope inclination” and “curvature” were derived from the digital elevation model with a 10 m spacing grid (DEM 10, Federal Agency for Cartography and Geodesy (BKG), 2016).

#### Copernicus land monitoring service

For information on land use and the quantification of soil losses caused by soil sealing in Germany two data products of the freely accessible European Union’s Copernicus Land Monitoring Service information (CLMS, European Union, 2023a), the CLC and the CLMS High-Resolution Imperviousness layers, are used.

The CLC 2006 (European Union, 2023b) and 2012 (European Union, 2023c) are applied for all calculations that depend on land use, i.e., the utilization of the BÜK 200 (see “Soil, climate and terrain data” section) and the determination of soil loss on agricultural land. Thereby, agricultural land use includes the CLC classes non-irrigated arable land (Code 211), vineyards (221), fruit trees and berry plantations (222), complex cultivation patterns (242) and land principally occupied by agriculture (243), and pastures (231) corresponding to cropland and grassland, respectively (European Environment Agency, 2021b).

The CLMS High-Resolution Imperviousness layers are available as status layers for five reference years 2006, 2009, 2012, 2015 and 2018, and as classified change layers (IMCC) comprising differences between status layers of those five years. In the present study only the IMCC layers 2006–2009 (European Union, 2023d), 2009–2012 (European Union, 2023e) and 2012–2015 (European Union, 2023f) are considered, because the reliability of the magnitude of imperviousness increase in 2018 and IMCC 2015–2018 (European Union, 2023g), respectively, was, at the time of writing, still being investigated by CLMS itself (European Union, 2023h). The rate of soil sealing in these layers is a product of automated derivation based on the calibrated normalized difference vegetation index (*NDVI*). The sealing change product IMCC has a resolution of 20 m and is separated into six different categories: unchanged no sealing, new cover, loss of cover, unchanged sealing, increased sealing, decreased sealing (European Environment Agency, 2018). To determine the quantitative and qualitative sealing on soil previously used for agriculture, only the areas of IMCC layers classified as new cover are investigated. For further analysis, they are intersected with areas under agricultural land use as indicated by the according CLC year: 2006–2009 and 2009–2012 using CLC 2006 and 2012–2015 using CLC 2012, respectively.

#### Federal statistical office data

In addition to the IMCC layers, soil sealing was also determined using the freely available area-use statistics of the Federal Statistical Office, both Germany-wide and with regional databases (Federal and State Statistical Offices, 2023c; Federal Statistical Office (Destatis), 2023). It is estimated that the majority of the disappeared agricultural land has been converted to predominantly sealed housing and transportation sector, and only a negligible amount has been restored. Therefore, the comparison in this study focuses on the official statistics data on the loss of agricultural soil.

The data of the German Main Land-use Survey (Federal Statistical Office (Destatis), 2022a) is used to determine the overall loss and therefore the approximate soil sealing of agricultural soils in Germany. To compare IMCC data with official statistics of Lower Saxony and Brandenburg, the changes in area by type of actual use between 2004 and 2015 are used to approximate the soil sealing on agricultural soils between 2006 and 2015, since only data from 2004 and subsequently 2008 are available (Federal and State Statistical Offices, 2023c). Due to size changes of administrative boundaries 2012/2013 in Brandenburg, areas are calculated separately from 2004 to 2012 and from 2013 to 2015. The year in between was interpolated.

For these two local test areas OLD and BER official statistics of the respective municipalities are used from the earliest available year 2008 to 2015 (Federal and State Statistical Offices, 2023c). The changes in loss of agricultural soils are area-weighted depending on the municipalities share in these two grid cells and subsequently added up to approximate the overall sealing rate for both 20,000 ha areas.

### Data analysis and visualization

All data analysis was conducted using the R software version 4.0.3 (R Core Team, 2020) and QGIS version 3.6.12 (QGIS Development Team, 2021). The soil assessment using the seven different soil functions and potentials as described above in “Soil functions andpotentials” section was realized using the R packages *sf* and *raster* (Hijmans, 2021; Pebesma, 2018). Calculations were only conducted for the agricultural area which is reported by the CLC data (see “Data collectionand data processing” section).

**Table 1:** Evaluation of soils under agriculture in Lower Saxony using scores from 1 (very low) to 5 (very high) or 1 (very low) to 6 (very high) (EW and EA) for seven different soil functions and potentials: erosion resistance against water (EA), erosion resistance against wind (EW), runoff regulation function (RR), mechanical filter function (MF), physical-chemical filter function (CF), biotic yield potential (BP) and Müncheberg Soil Quality Rating (SQR). They are presented as absolute area and percentage of the agricultural area of Lower Saxony according to CLC 2012

**Score**		**1**	**2**	**3**	**4**	**5**	**6**
**EA**	**km²**	951	1017	707	478	6116	22,515
	**%**	3.0	3.2	2.2	1.5	19.1	70.4
**EW**	**km²**	599	1528	4965	5418	6751	3588
	**%**	1.9	4.8	15.5	16.9	21.1	11.2
**RR**	**km²**	0	10	13,943	15,802	0	−
	**%**	0.0	0.0	43.6	49.4	0.0	−
**MF**	**km²**	154	901	1966	7908	19,994	−
	**%**	0.5	2.8	6.1	24.7	62.5	−
**CF**	**km²**	8958	9546	6644	5927	308	−
	**%**	28.0	29.9	20.8	18.5	1.0	−
**BP**	**km²**	11,139	8030	4352	5746	515	−
	**%**	34.8	25.1	13.6	18.0	1.6	−
**SQR**	**km²**	313	12,231	10,851	5580	276	−
	**%**	1.0	38.2	33.9	17.4	0.9	−

Each of the soil functions and potentials has a different set of input variables (see Fig. 9 in the Appendix). Depending on the soil data availability in the BÜK 200 the percentage of the evaluated area can vary from soil function to soil function. It can happen that for certain soil regions a determination is not possible due to a missing input variable and therefore the evaluated area does not cover all the agriculturally used land (100%) but, for example, only reaches 71%.

The density distribution was calculated and displayed using violin plots and boxplots of the *ggplot2* package (Wickham, 2016). To visualize and compare the quantitative soil loss for Lower Saxony and Brandenburg the federal states were divided into a mesh with a grid size of $10\,\times 10$ km.

## Results

### Evaluation of soil functions and potentials

The soil functions and potentials were determined for the two consecutive available CLC years 2006 and 2012. Since the results of both years have largely similar patterns, in the following the more recent scores from 2012 are displayed for the federal states of Lower Saxony and Brandenburg.

**Fig. 2:**
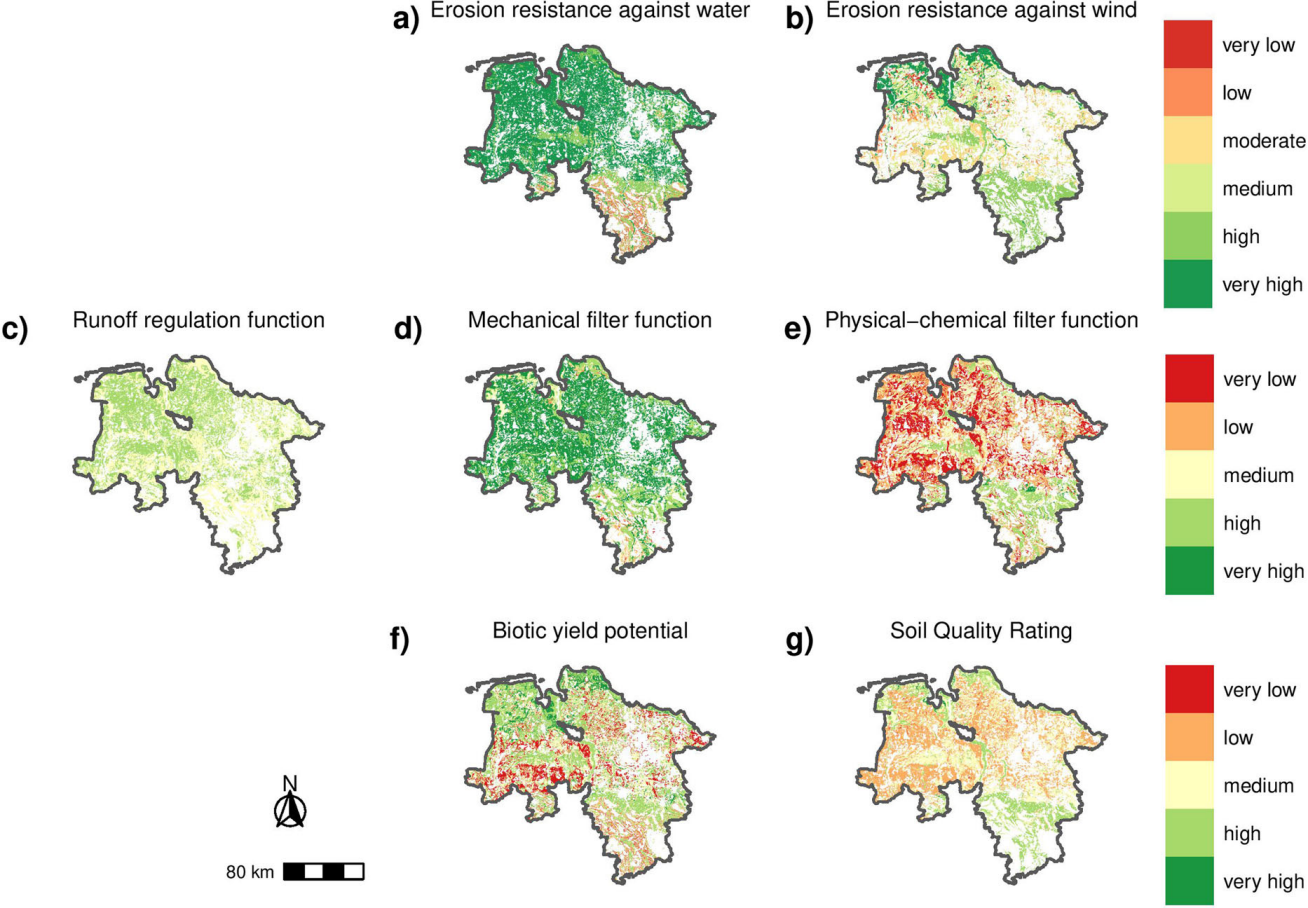
Evaluation of soil functions and potentials for Lower Saxony based on the land use of CLC 2012

#### Federal state of Lower Saxony

Lower Saxony covers an area of 47,614 km^2^ of which 31,980 km^2^ were used for agriculture in 2012 according to the CLC. Of this area, only a total of 71% can be evaluated in terms of erosion resistance against wind, however all of the other six functions exceed 90% coverage of agricultural use (Table 1). The soil functions of erosion resistance against water, runoff regulation and mechanical filter (Fig. 2a, c, d) are all evaluated as high to very high for whole Lower Saxony except for the southernmost part of the state and the more mountainous regions of Lower Saxony, namely the Harz mountains and the Harz foothills as well as the extensions of the Lower Saxony and Hesse uplands. An inverted picture is visible for erosion resistance against wind, physical-chemical filter function and SQR (Fig. 2b, e, g), where moderate to very low scores are determined for the most parts of Lower Saxony. Merely the coastal regions and the southern part are rated with high to very high scores. Biggest differences in the soil evaluation are evident for the biotic yield potential (Fig. 2f). In general, the potential is medium to high for the entire area, with especially high ratings in the grassland regions at the coast as well as the loess soils of Brunswick and Hildesheim. However, the sandy soils and podzol soils under heathland partially reach only very low scores.

**Table 2:** Evaluation of soils under agriculture in Brandenburg using scores from 1 (very low) to 5 (very high) or 1 (very low) to 6 (very high) (EW and EA) for seven different soil functions and potentials: erosion resistance against water (EA), erosion resistance against wind (EW), runoff regulation function (RR), mechanical filter function (MF), physical-chemical filter function (CF), biotic yield potential (BP) and Müncheberg Soil Quality Rating (SQR). They are presented as absolute area and percentage of the agricultural area of Lower Saxony according to CLC 2012

**Score**		**1**	**2**	**3**	**4**	**5**	**6**
**EA**	**km²**	28	109	138	274	2898	11,763
	**%**	0.2	0.7	0.9	1.8	19.0	76.9
**EW**	**km²**	65	278	5042	1809	1749	1043
	**%**	0.4	1.8	33.0	11.8	11.4	6.8
**RR**	**km²**	0	0	3144	10,448	0	−
	**%**	0.0	0.0	20.6	68.3	0.0	−
**MF**	**km²**	0	62	245	1537	11,431	−
	**%**	0.0	0.4	1.6	10.1	74.8	−
**CF**	**km²**	4193	3900	2163	3217	125	−
	**%**	27.4	25.5	14.2	21.0	0.8	−
**BP**	**km²**	876	1630	7033	4683	649	−
	**%**	5.7	10.7	46.0	30.6	4.2	−
**SQR**	**km²**	17	3849	7597	1489	0	−
	**%**	0.1	25.2	49.7	9.7	0.0	−

#### Federal state of Brandenburg

The federal state of Brandenburg covers around 29,479 km² with roughly half of it being agriculturally used according to CLC 2012 (15,287 km^2^). Similarly to Lower Saxony, erosion resistance against wind contains the smallest area of calculated soil function that only evaluates 65% of the agricultural land (Table 2). This is contrasted by the remaining soil functions and potentials, which reach a coverage between 85% (SQR) up to 99% (erosion resistance against water).

**Fig. 3:**
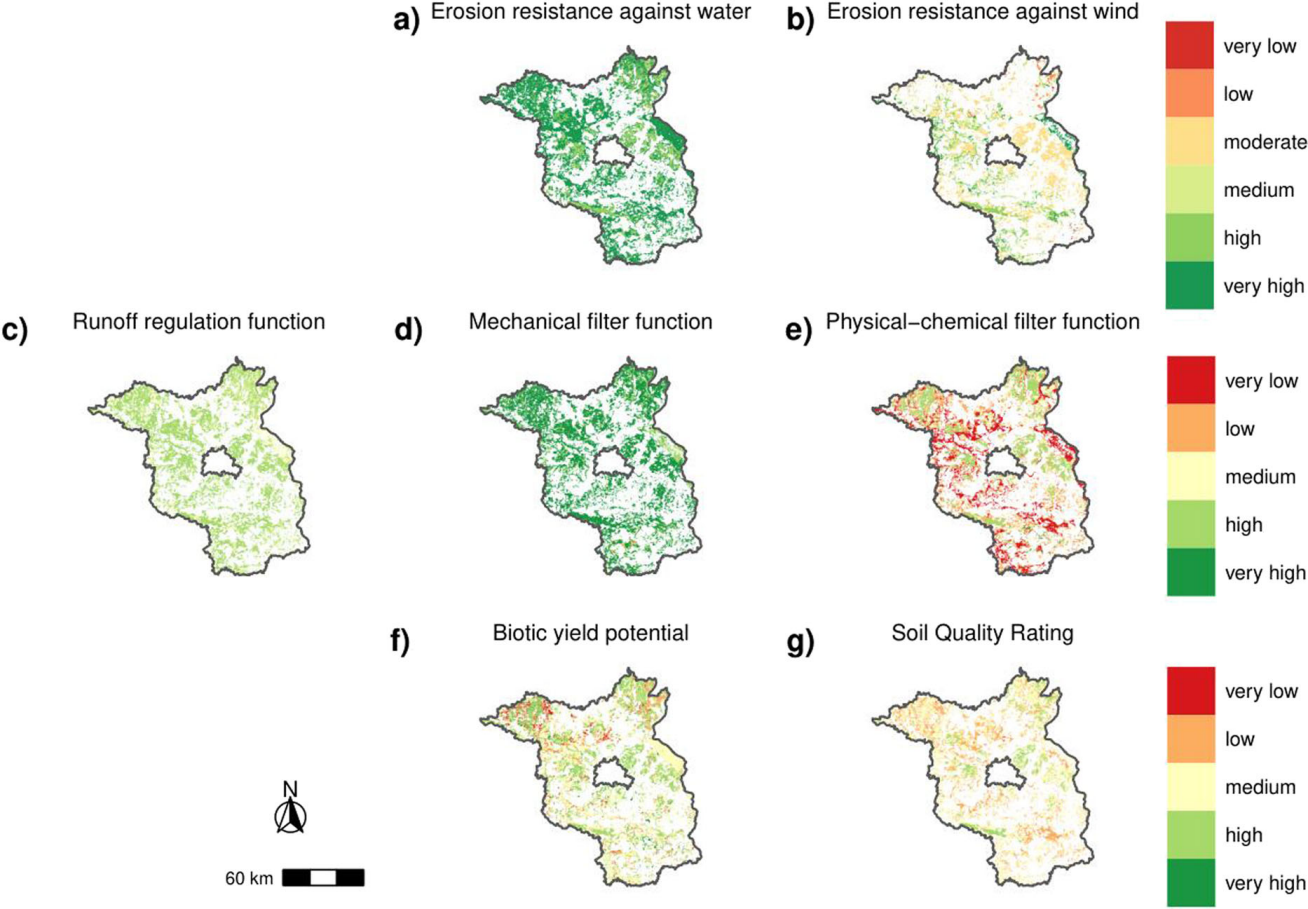
Evaluation of soil functions and potentials for Brandenburg based on the land use of CLC of the year 2012

The agricultural soils of Brandenburg have very high ratings for their erosion resistance against water and mechanical filter function and medium to high scores for the runoff regulation function (Fig. 3a, c, d). The other four functions and potentials show mixed results from high to very low with lowest evaluations for the physical-chemical filter function (Fig. 3b, e, f, g). Results of the biotic yield potential (Fig. 3f) display medium values for the whole federal state with higher evaluated soils in the regions of Fläming (southwest), Prignitz (northwest), Uckermark (northeast), Havelland (west of Berlin) and Barnim (east of Berlin). The SQR (Fig. 3g) results show overall lower scores compared with the biotic yield potential, however the regions with better ratings are similar.

**Fig. 4:**
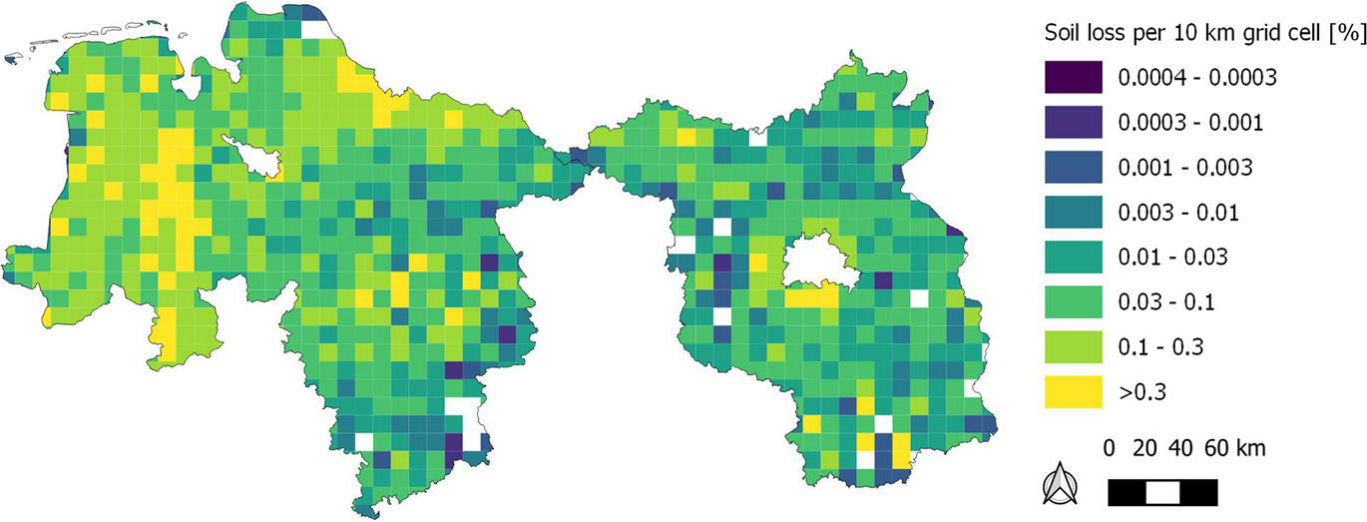
Accumulated sealed agricultural soils of Lower Saxony and Brandenburg from 2006 to 2015 presented as percentages of lost agricultural soils in a $10\times 10$ km grid

### Quantitative soil loss

#### Federal state of Lower Saxony

For Lower Saxony, the soil sealing rate for the three time steps of three years each is 3.02, 2.35 and 0.35 ha day^-1^ for 2006–2009, 2009–2012 and 2012–2015, respectively (Table 3).

In the 10 km grid-based Fig. 4, the accumulated amounts of agricultural soil loss between 2006 and 2015 are displayed. It is clearly visible that Lower Saxony has higher losses of agricultural soil between 2006 and 2015 than Brandenburg. This applies both to the representation per 10 km grid cell and to the total area amount. Lower Saxony lost 6269 ha, i.e., 1.9 ha day^-1^ of agricultural soil between 2006 and 2015 (Table 3). Regions with the highest quantity of soil loss are generally visible in the surroundings of bigger cities like Hamburg and Hanover. However, a considerate amount of soil is lost in more sparsely populated and rural regions as well, for example, in the western parts of Lower Saxony (Fig. 4). In grid cells with increased soil loss, these soil sealings often occur as a conglomerate of small or medium sized sealings consisting mainly of roads, housing and commercial sites (data not shown).

**Fig. 5:**
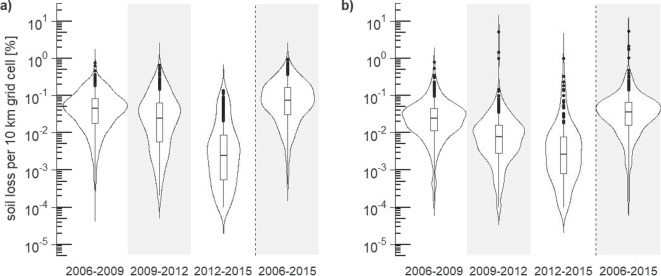
Density distributions and boxplots of calculated percentages of soil losses of agricultural soils in a $10\times 10$ km grid of (a) Lower Saxony and (b) Brandenburg separated into the three time periods 2006–2009, 2009–2012 and 2012–2015 as well as the accumulated time period 2006–2015 as presented in Fig. 4

When comparing the statistical characteristics of the three different time sections, differences in density and distribution of the data are visible (Fig. 5). Overall, for both Lower Saxony and Brandenburg, the time period of 2006–2009 shows the highest values of all three sections with the smallest interquartile ranges. Lower values are disclosed for the two following time periods 2009–2012 and 2012–2015, however at the same time the data shows a wider dispersion. During the complete period 2006 to 2015 Lower Saxony had a median of 0.07% (Fig. 5a). Between 2006 and 2009, the interquartile range extends from 0.02 to 0.08% with a median of 0.04%. In the time period of 2009–2012, the interquartile range comprises 0.006 to 0.063% with a median of 0.02%. It is clearly visible that the last time period 2012–2015 features less and even smaller values of sealed surfaces than the previous two time periods with 0.0006 to 0.0009% and 0.0025% being the interquartile range and the median, respectively. While the minimum values are similar for all three time periods, the maximum boxplot value of the time period 2012–2015 (0.02%) corresponds with the medians of the previous two time periods. The boxplot maxima being 0.18% and 0.14% for the time periods 2006–2009 and 2009–2012, respectively.

**Table 3:** Quantitative soil loss of agricultural soils according to the Imperviousness Classified Change layers (IMCC) and federal and state Statistical Office data for Germany, Brandenburg, Lower Saxony, Berlin-Airport (BER) and Oldenburg (OLD)

		**IMCC**				**Statistical Office**
		**’06–’09**	**’09–’12**	**’12–’15**	**’06–’15**	2006–2015*
**Germany**	**ha**	19,542	15,010	2422	36,974	260,000
	**ha day** ^-1^	17.85	13.71	2.21	11.26	79.15
**Brandenburg**	**ha**	1243	1116	379	2738	5600
	**ha day**^-1^	1.14	1.02	0.35	0.83	1.70
**Lower Saxony**	**ha**	3310	2574	385	6269	28,800
	**ha day**^-1^	3.02	2.35	0.35	1.91	8.77
**BER**	**ha**	60	654	17	731	1030
	**ha day**^-1^	0.05	0.60	0.02	0.22	0.40
**OLD**	**ha**	87	79	7	172	225
	**ha day**^-1^	0.08	0.07	0.01	0.05	0.09

*2008–2015 for BER and OLD

#### Federal state of Brandenburg

Soil sealing rates for Brandenburg are 1.14, 1.02 and 0.35 ha day^-1^ for the three time steps of three years, 2006–2009, 2009–2012 and 2012–2015, respectively, and are therefore clearly smaller than rates of Lower Saxony (Table 3).

The accumulated amounts of agricultural soil loss in Brandenburg between 2006 and 2015 are 2738 ha or 0.8 ha day^-1^ (Fig. 4b, Table 3) which is clearly lower than the soil loss in Lower Saxony. The highest quantity of soil loss occurred in the surroundings of Berlin. The grid cells with more than 0.3% of soil loss in general are mostly caused by solar parks in the southern and north-western part of Brandenburg, the newly built airport Berlin-Brandenburg, and infrastructure and housing south of Berlin (Fig. 4). In grid cells with increased soil loss, these soil sealings often occur as larger, contiguous areas in Brandenburg (data not shown).

As already mentioned in “Federal state of LowerSaxony” section there are clear differences between the times sections in terms of density and distribution which are similar for Lower Saxony and Brandenburg. The interplay of shrinking size of the interquartile range, wider dispersion of the data, and generally lower soil losses from 2006–2009 to 2012–2015 is particularly evident in Brandenburg (Fig. 5b). Overall from 2006 to 2015 the median of Brandenburg (0.04%) is lower than the median of Lower Saxony.

**Fig. 6:**
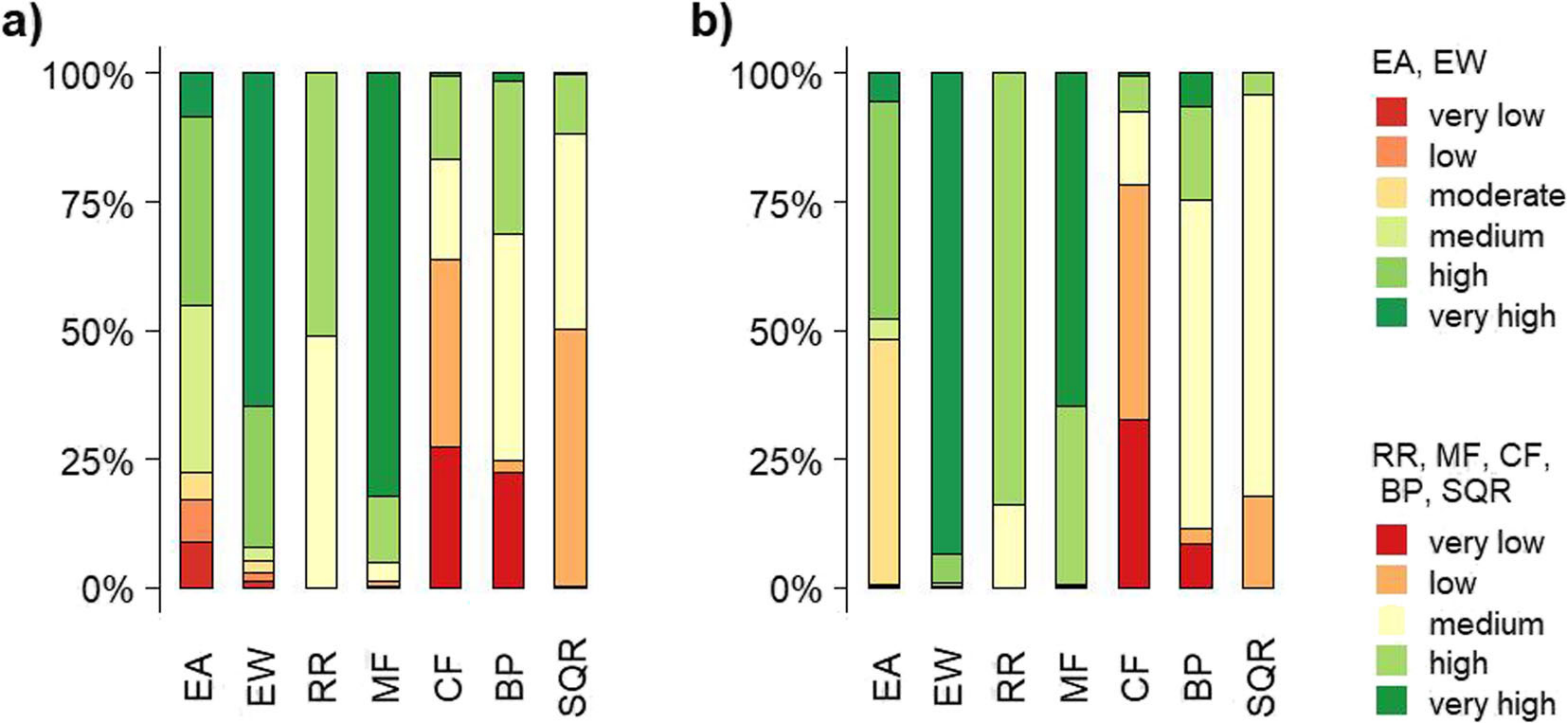
Percentage shares of the previous soil functions and potentials of sealed agricultural soils divided into assessment classes for (a) Lower Saxony and (b) Brandenburg shown cumulatively between 2006 and 2015. EA: erosion resistance against water, EW: erosion resistance against wind, RR: runoff regulation function, MF: mechanical filter function, CF: physical-chemical filter function, BP: biotic yield potential, SQR: Müncheberg Soil Quality Rating

The data distribution for Brandenburg shows interquartile ranges from 0.01 to 0.05%, 0.0003 to 0.0158% and 0.0008 to 0.0077% for the time periods 2006–2009, 2009–2012 and 2012–2015, respectively. Medians reach higher values in the first two time periods with 0.024 and 0.008%, while the lowest median is determined as 0.003% between 2012 and 2015.

#### Federal and state statistical office data

According to the German Main Land-use Survey, the land area under agricultural use decreased by approximately 260,000 ha between 2006 and 2015 (Table 3). The official statistics show 5600 ha and 28,800 ha of losses in agricultural soils for Brandenburg and Lower Saxony, respectively. Estimates of soil loss on agricultural land between 2008 and 2015 for the two local scale test area grid cells around Oldenburg (OLD) based on seven involved municipalities show a decrease of 225 ha. The amount of agricultural soil loss for the surroundings of the Berlin-Brandenburg Airport (BER test area) reaches a total of 1030 ha between 2008 and 2015.

### Qualitative soil loss

#### Federal state of Lower Saxony

The soil evaluations of sealed soils in Lower Saxony and Brandenburg show major losses on medium or better rated soils (Fig. 6). In Lower Saxony, between 0% (runoff regulation function, RR) and 63% (chemical-physical filter function, CF) of all sealed soil between 2006 and 2015 are assessed as very low or low in their soil functions and potentials, whereas between 12% (SQR) and 95% (mechanical filter function, MF) are rated as high or very high (Fig. 6a). Roughly three quarters of all newly sealed soils in Lower Saxony between 2006 and 2015 show a medium, high or very high biotic yield potential (BP), and half of the them are rated medium or high in terms of the SQR.

**Fig. 7:**
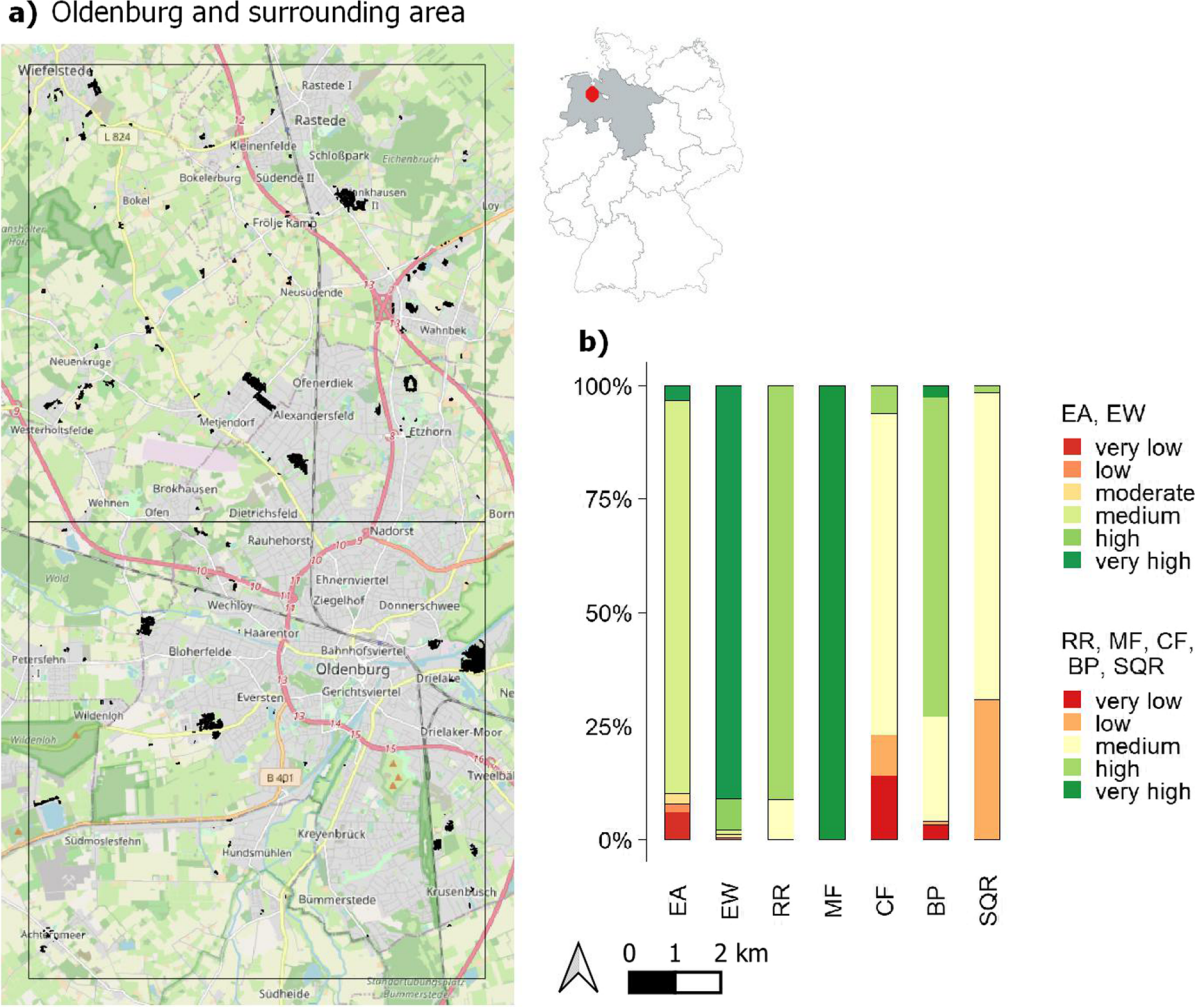
(a) Cumulative sealed areas between 2006 and 2015 (IMCC) at Oldenburg and the surrounding area in two $10\times 10$ km grid cells adding up to 172 ha (hashed black areas), and (b) mean values of the previous soil functions and potentials of the sealed agricultural soils. EA: erosion resistance against water, EW: erosion resistance against wind, RR: runoff regulation function, MF: mechanical filter function, CF: physical-chemical filter function, BP: biotic yield potential, SQR: Müncheberg Soil Quality Rating. OpenStreetMap is used as map base in (a) (OpenStreetMap, 2023)

#### Federal state of Brandenburg

For Brandenburg, on the one hand, between 0% (runoff regulation function, RR) and 78% (chemical-physical filter function, CF) of the soils are rated as very low or low, on the other hand, between 8% (chemical-physical filter function, CF) and 99% (erosion resistance against wind, EW) are evaluated as high or very high concerning their soil functions and potentials (Fig. 6b). The SQR and the biotic yield potential (BP) are rated as medium or better for 82% and 88% of the sealed soils, respectively. The overall small shares of poorly rated soils or the large shares of well rated soils in relation to erosion resistance against wind (EW), runoff regulation function (RR) and mechanical filter function (MF) are due to nationwide good ratings for these soil functions overall in both federal states (see Figs. 2 and 3).

#### Local scale test areas

A more detailed investigation of the sealed soils in Lower Saxony around Oldenburg (OLD test area) in two $10\times 10$ km grid cells shows that the newly sealed soils of the IMCC layers add up to 172 ha (Fig. 7a, Table 3) between 2006 and 2015. Slightly less than half of the sealed soils can be approximately attributed to the commercial sector (47%), while the other half was sealed for housing purposes (53%; data not shown).

**Fig. 8:**
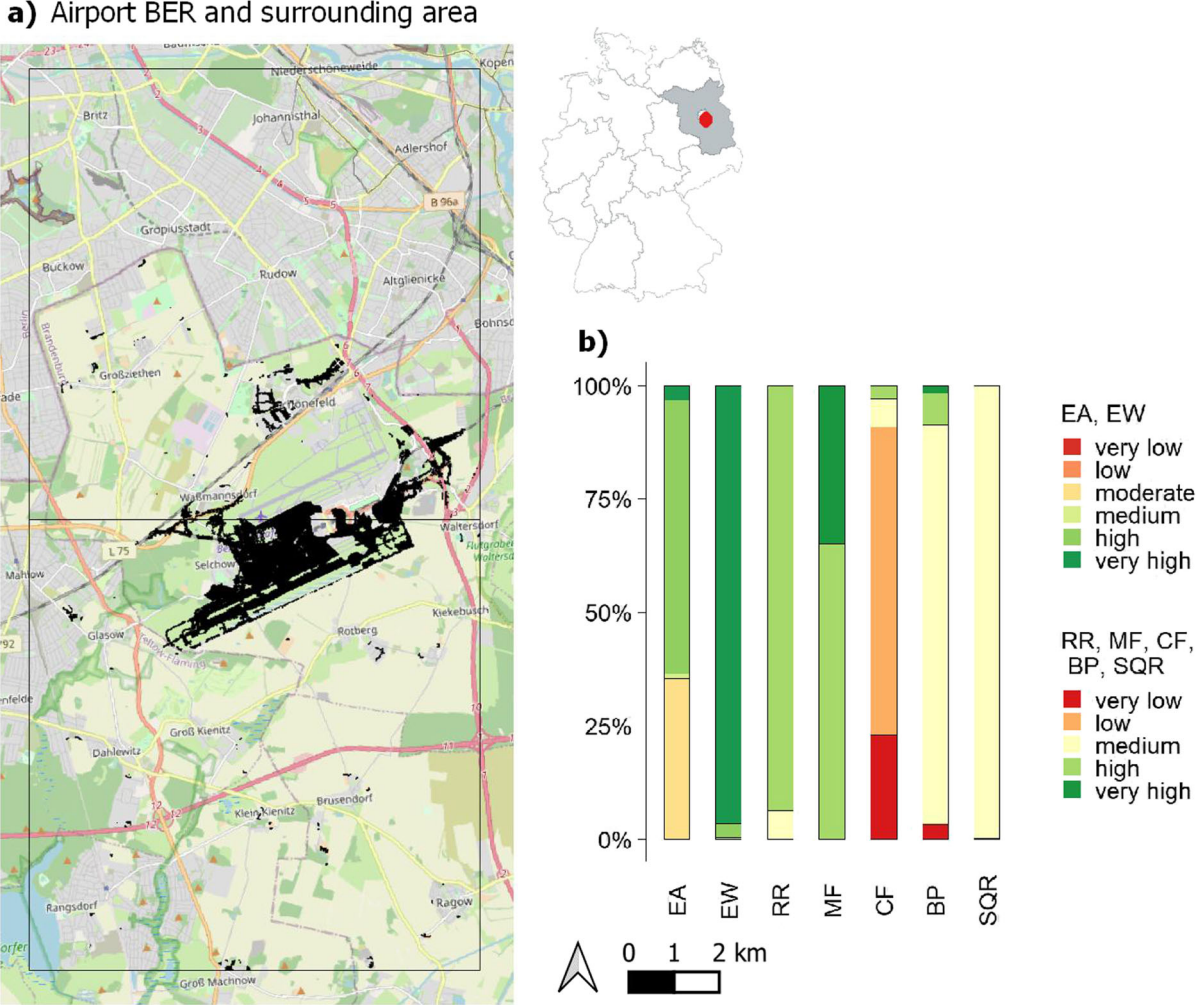
(a) Cumulative sealed areas between 2006 and 2015 (IMCC) at Berlin-Brandenburg Airport (BER) and the surrounding area in two $10\times 10$ km grid cells adding up to 731 ha (hashed black areas), and (b) mean values of the previous soil functions and potentials of the sealed agricultural soils. EA: erosion resistance against water, EW: erosion resistance against wind, RR: runoff regulation function, MF: mechanical filter function, CF: physical-chemical filter function, BP: biotic yield potential, SQR: Müncheberg Soil Quality Rating. OpenStreetMap is used as map base in (a) (OpenStreetMap, 2023)

The sealed soils in the OLD test area predominantly have medium, high or very high rated soil functions and potentials. This is again partly caused by nationwide good rating of specific soil functions as previously mentioned (Fig. 7b). Only biotic yield potential (BP), erosion resistance against water (EA) and physical-chemical filter function (CF) have very low ratings with 3, 6 and 14%, respectively. The SQR (31%), physical-chemical filter function (CF, 9%) and erosion resistance against water (EA, 2%) moreover show considerate shares of soils with low ratings. The soil evaluations show better rated soil functions and potentials for the soil under housing areas than for soils under commercial areas, implying that more valuable soils are lost for housing purposes for this example of Oldenburg and surroundings (data not shown).

A closer look at IMCC soil sealings associated with the Berlin-Brandenburg Airport (BER test area) and its surrounding area in Brandenburg reveals 731 ha of soil loss between 2006 and 2015 (Fig. 8a, Table 3). Compared to soil losses on agricultural land caused by the commercial (6%) and infrastructure sector (93%), the housing is only accountable for 1% in this area (data not shown).

The soil sealings show that out of all soil functions and potentials, four only have soil ratings that are of medium or higher scores (Fig. 8b). In contrast, 35% of the sealed soils have moderate erosion resistance against water (EA), 91% have low or very low physical-chemical filter function (CF), and 3% have very low biotic yield potential (BP). Soil losses caused by the construction of the new airport and adjacent infrastructure account for most of the soil sealing in the area, and the distribution is accordingly. The soil evaluation under newly sealed commercial areas displays high percentages of low or very low rated physical-chemical filter function (CF) and biotic yield potential (BP), but at the same time 25% of very high rated BP. Even though the housing sector is only accountable for 1% in this area, the occurrence and allocation of medium or higher rated soil functions and potentials is overall comparable or even of a slightly higher frequency than for commercial sector soil sealings (data not shown).

### Statistical office vs. IMCC data

For Germany-wide quantitative soil loss, which considers only agricultural soils, the IMCC data are 6.5 times smaller than the statistics, resulting in values that are 85% lower than the official statistics Table 3. Soil loss determined from IMCC data is 4.5 times lower in Lower Saxony and two times lower in Brandenburg than official statistics when soil sealing on agricultural soils is taken into account, i.e., the values are 78% and 51% lower, respectively, than official statistics. Lower Saxony’s share is relatively close to the order of magnitude for Germany as a whole, while Brandenburg’s share is clearly smaller. The lowest discrepancies were determined in the local scale test areas in the two 10 $\times$ 10 km grid cells. Remote sensing IMCC data showed 25% lower values than official statistics for the OLD test area and 30% lower values for the BER test area. However, the official data of the municipalities only goes back to 2008, as the earlier capture date of 2004 is not available for download. Thus, the actual loss of agricultural soil will be even higher, and the differences between IMCC and statistics are likely to be slightly greater.

## Discussion

### Soil evaluation

#### Methods for soil evaluation

The holistic approach used in the study is important for a comprehensive assessment of soil quality (Bünemann et al., 2018), especially since the literature often ignores extrinsic factors such as site-specific characteristics, management or climatic data (e.g., Armenise et al., 2013; Cotching and Kidd, 2010; Juhos et al., 2019, Santos-Francés et al., 2019). With the approaches of Marks et al. (1992) and Müller et al. (2007) the input variables in the present study are broadly based and the determined soil functions and potentials assess a majority of the soil ecosystem services. Although in this study we have made an effort to include and consider as much intrinsic and extrinsic information as possible, there are still influencing variables that have not yet been incorporated such as biological indicators or soil and crop management. However, for example, biological indicators are missing in 40% of all reviewed soil assessments by Bünemann et al. (2018), although its importance is well known, they still require specific knowledge and skills and measurement which is time consuming and costly.

Different aggregation approaches can be used to condense the information that are assembled by multiple indicators or functions into one general soil quality index. Typically, aggregation methods include summarizing of all indicators (Andrews et al., 2004; Velasquez et al., 2007). An improvement can be achieved by including constructive weighting factors that are based on the importance of the respective soil function or ecosystem service for the overall evaluation goal (Armenise et al., 2013; Klimkowicz-Pawlas et al., 2019; Liu et al., 2020; Müller et al., 2007). However, the aggregation of soil functions and potentials was omitted in the present study in order to allow for more transparency and to leave options open to the user, such as the combination of data or the further use of those. Given the fact that each displayed soil function already comprises multiple input variables, one aggregated index carries the risk of overgeneralizing the informational content of the soil evaluation.

Even though there are numerous previous soil quality assessments in the literature, the majority of studies focuses on a single plot or site only (e.g., Askari and Holden, 2014; Lima et al., 2013; Rutgers et al., 2012; Shukla et al., 2006). Of course, a soil quality index that can be applied everywhere cannot be created, as there are significant local differences all over the world. However, for similar conditions, it should be possible to use assessment methods on a larger scale, under the important prerequisite that the methods are standardized and they do not have spatial scale related problems (Bastida et al., 2008). Accordingly, well-documented approaches can be implemented for various soils and regions and provide an evaluation of soils in terms of specific soil functions, services or threats. The two methods used in this study, the evaluation of landscape ecosystem performance (Marks et al., 1992) and the Müncheberg Soil Quality Rating (SQR, Müller et al. 2007), were developed for use on a larger scale. The here presented results are indicative for the good reliability of the two evaluation systems. There were comprehensible and partly already known regions with better or worse evaluations of certain soil functions, such as the sandy Cambisols or Histosols compared to regions with fertile Chernozems and Phaeozems or Luvisols formed on the loess belt, or the difference between coastal and mountain regions in Lower Saxony (Altermann et al., 2005; Poeplau et al., 2020; Richter et al., 2009; IUSS Working Group WRB, 2022). Furthermore, there was a general agreement between the results of the biotic yield potential and the SQR which was expected due to similar initial variables.

#### Alternative sources for input variables

With the soil evaluation applied in this study, a lot of different input variables were used. This concerns soil information including chemical and physical properties, but also climate, land use and site-specific data. The data are characterized by different geometric and semantic resolutions and thus by different scale-specific explanatory power (Möller & Volk, 2015). One approach to reduce or resolve the scale-specific discrepancies is to aggregate the results on small-scaled reference units (Volk et al., 2010), as shown in Fig. 4. Currently, statewide or regional Digital Soil Mapping products are also being generated for Germany (e.g., Broeg et al., 2023; Gebauer et al., 2022; Möller et al., 2022; Sakhaee et al., 2022; Zepp et al., 2021) that may act as nationwide data bases in the future.

In Germany, there is a multitude of different soil data sets at different spatial scales. The highest resolution nationwide soil map with consistent information on soil conditions across all federal states is the BÜK 200, which was utilized in this study to derive soil functions, potentials and hazards. However, it was not straightforward to make use of BÜK 200, but required the development of a previously non-existent complex workflow, which was described in detail in “Soil, climate and terrain data” section. This workflow can now be deployed and adapted to make use of the BÜK 200 for various other application fields.

One important input variables to utilize the BÜK 200 data set is land use information. The “Amtlich Topographisch-Kartographische Informationssystem” (ATKIS; Arbeitsgemeinschaft der Vermessungsverwaltungen der Länder der Bundesrepublik Deutschland (AdV) n.d.) is a conventional land use information source. The ATKIS data has the disadvantage that the land use information is only updated irregularly and the spatial resolution is rather coarse. Another possibility, especially for the analysis of soil loss and respective soil evaluations from 2018 onwards, could be the data set “Digitales Landbedeckungsmodell für Deutschland” (LBM-DE 2018; Federal Agency for Cartography and Geodesy (BKG) 2018). The LBM-DE dataset is a hybrid of remote sensing data and survey data that is based on selected areal object types of the ATKIS Basis-DLM from the sectors settlement, transportation, vegetation and water bodies, which have been modified to the specific requirements of the CORINE Land Cover (CLC). The main application of the LBM-DE is the derivation of the CLC for the area of Germany. The advantages of LBM-DE for the future include its higher resolution compared to CORINE CLC, consideration of only relevant CLC categories occurring in Germany are considered, and higher accuracy due to the use of additional data such as other satellite images and digital aerial images to allow more precise interpretations or to correct errors. In this study, the CLC was used, which is freely and openly accessible via the Copernicus Land Monitoring Service (CLMS) for multiple years and has a sufficient minimal mapping unit of 25 ha or 100 m width. The decisive advantages for the use of CLC is the simple acquisition of all previously available time stamps back to 2006, the detailed documentation of the data and the standardized mapping for whole Germany.

The Integrated Administration and Control System (IACS) serves as basis for mangement of agricultural funds from the European Union and contains land use and crop type information. The IACS data is available every year, however, the completeness or correctness cannot be conclusively clarified, as reporting by farmers is voluntary. Moreover, the IACS data is not freely available Germany-wide. A future possible alternative to IACS and to land use information in general could be provided by satellite-based crop type classifications (e.g., Asam et al., 2022; Blickensdörfer et al., 2022; Preidl et al., 2020). For the analyses carried out here, however, a distinction between arable land and grassland was sufficient.

It is important to point out that the soil evaluation, although processed and presented in a high resolution of $10\times 10$ m, is relying on data of coarser resolution of up to $1\times 1$ km (see “ Data collection and data processing” section). However, as pointed out in the methods, the soil evaluation is majorly dependent on the soil information based on the BÜK 200, thus the resolution is ultimately based on the scale of 1:200,000. The goal of this study was to evaluate all of Germany, so the BÜK 200 was used as the only available comprehensive Germany-wide soil map. Using higher resolution soil data from German federal states would not have been purposeful, as it would only result in a patchwork due to the different data sets and mapping bases. These limitations of higher resolution data can only be overcome if soil data are harmonized across all federal states in the future. Nevertheless, the significant differences of soil quality of Lower Saxony and Brandenburg as well as in Germany in general, disclose the necessity of site-specific or area-specific soil evaluations by region. Thereby using higher resolution soil data of the federal states, for example, 1:50,000, and an adjusted rating system to evaluate smaller regions is beneficial as demonstrated in Engel and Stadtmann (2020).

### Soil sealing

#### Importance of precise location and quality

Germany’s actions on land degradation and land consumption is outlined in the National Sustainable Development Strategy of Germany, which includes the ambitious goals of the limiting and reduction of additional sealing for settlement and transport to an average of 30 ha day^-1^ until 2030, and furthermore to stop sealing soil for settlement and transport purposes altogether by 2050 (Bundesregierung, 2016). Even though the total extent of soil sealing in Germany is known and documented (Federal Statistical Office (Destatis), 2022b, a, 2023), there is still a lack of information on the exact location and past soil quality of sealed soils. Ongoing changes in population size and behavior as well as in infrastructure and tourism sectors are causing (sub)-urbanization and new infrastructure constructions. This is of particular concern given that sealed soils are consuming some of the best agricultural soils. Urban expansion alone could lead to the loss of 2% of the world’s arable land by 2030, which is on average 1.8 times more productive land than the global level (Bren d’Amour et al., 2017). Cities have historically been built on fertile land, and urban centers often expand on the most productive land (Satterthwaite et al., 2010). European cities are becoming more dispersed as low-density settlements spread (urban sprawl) (Kasanko et al., 2006). This is also visible in the local test area of the Berlin-Brandenburg Airport, or Lower Saxony where nearly 75% had medium or higher biotic yield potential. This situation leads to a forced, inevitable shift of agriculture to maybe less productive land in Germany or even outsourcing production to other countries. A study of urban development in Greece came to a similar conclusion, where soil sealing selectively consumed the best soils available in the study area (Nickayin et al., 2021). As the examples of Lower Saxony and Brandenburg have shown, a functioning and relatively accurate estimation of the quality of the used soil is possible with the approach presented here by combining spatial information on soil sealing with the Imperviousness Classified Change Layers (IMCC) layers and the evaluation of the soil using the BÜK 200.

#### Discrepancies between statistical office and IMCC data

The large discrepancies between official area survey data and remote sensing soil loss quantity point towards a lower accuracy of the IMCC data and indicate that the soil sealing reported by the IMCC layers is most likely substantially underestimating the actual amount. It must be emphasized, however, that this study was not designed as a credibility check of the IMCC data, but to use novel remote sensing data products along with soil evaluation to show its potential for qualitative and quantitative assessment of the loss of agricultural soils. It is not a systematic comparison of official administrative data and remote sensing data. Nevertheless, in a study analyzing the content and accuracy of the High-Resolution Layers as status layer (IMD) from 2018 in Norway, the IMD sealing data were overall around 40% below the official sealing given by Statistics Norway and 33% below the estimate based on aerial photographs (Strand, 2022). The study of Strand (2022) and two studies in Poland and Slovakia, which were using the predecessor of the HRL layers, showed inaccuracies regarding on the one hand an overestimation of imperviousness in built-up, urban areas and an unsystematic omission of imperviousness in rural areas with sporadic soil sealing on the other hand (Congedo et al., 2016; Strand, 2022; Hurbanek et al., 2010; Krówczyńska et al., 2016). According to the EEA validation report, the user’s accuracy for all three time steps is just under 50%, while the producer’s accuracy for the 2009–2012 and 2012–2015 time periods decreases drastically compared to 2006–2009, showing an increase in omissions of sealed surfaces (Copernicus & European Environment Agency, 2019). The differences between the three time periods were also clearly visible in our results, especially for the period 2012–2015. Despite these inaccuracies and even though there is a considerable difference in accuracy for the studied time series from 2006 to 2015, these high-resolution imperviousness layers are currently the best way to accurately and in detail locate the soil loss required to evaluate the soil beneath sealed surfaces nationwide.

The settlement and transportation areas are often incorrectly considered as sealed surfaces although parks, gardens and open spaces within are also included. However, in fact sealed areas are surfaces that are built over or paved, i.e., asphalted or concreted surfaces (Frie & Hensel, 2009). Less than half (45.4% Germany-wide in 2015; Federal and State Statistical Offices 2023b) of the sealed soils in the housing and infrastructure sector are actually sealed. Based on this information, it can be assumed that the actual soil consumption on agricultural land is roughly twice the determined IMCC soil sealing since only actually sealed, impervious areas are included in these layers. In this context, actual soil consumption means that these areas are not used in their previous land use anymore. Thus, if this percentage of 45.4% of actually sealed soils is related to the total agricultural area in Germany, the IMCC data is only 67% instead of 85% below the official statistics. Along the lines, the IMCC data are 49% and merely 2.5% below the official statistics for Lower Saxony and Brandenburg, respectively and the IMCC data provide even higher amounts of sealing than the statistics for the Oldenburg (OLD) and Berlin-Brandenburg Airport (BER) test areas. Nevertheless the uncertainties of detecting impervious surfaces could be due to insufficient spatial resolution of the IMCC data to determine land loss at municipality level (Congedo et al., 2017).

It is quite surprising that the soil sealing amount is clearly in better agreement for Brandenburg compared with Lower Saxony, although the accuracy of IMCC data was very similar for both local scale test areas (Table 3). One reason could be the different sizes of imperviousness areas determined by the IMCC layers, given that the area size of the individual sealings is larger in Brandenburg than in Lower Saxony. It is also interesting to note that the discrepancies in the IMCC decrease as one moves down the scale from federal to state to regional. With the two test areas, two completely different settings were analyzed. On the one hand, the land around the newly constructed BER which is dominated by connected sealing and, on the other hand, a combination of rural and urban pattern and overall a more scattered behavior of the sealed areas in the surroundings of Oldenburg. This was also visible in the differing proportions of the sectors transportation, housing and commercial use (data not shown).

#### Future potential of IMCC data

Since the recorded statistical data of the type of actual use only allow to determine the total amount of soil sealing in a certain time frame, conclusions about the previous soil quality of this piece of land is therefore impossible. As shown in the present study, with the help of satellite-based data from the CLMS soil sealing can be quantified and spatially located. Remote sensing data methods and processing have improved significantly in recent years in terms of resolution and accuracy. For the IMCC layers, the improvement is clear for the years 2015–2018, where a higher resolution data time series based on Sentinel-1 and Sentinel-2 is used for the first time (time period not used in the present study, see “Copernicus land monitoring service” section). In this case, the resolution increases from 20 m to 10 m, and along with this, a significant increase in soil sealing has already been detected. Data from 2018 is clearly better in accordance with official statistics on soil sealing in Norway (Strand, 2022) than our results. Even though the IMCC data are standardized and covering the whole of Europe, the quality and accuracy varies per country. For Germany, there has been a decrease in user’s accuracy for the IMCC 2015–2018, but at the same time an increase in producer’s accuracy, according to the official validation report (Copernicus & European Environment Agency, 2020). Once the reliability of the magnitude of imperviousness increase of the IMCC layer from 2018 is investigated by the CLMS (European Union, 2023h), the analyses in this paper could be updated and improved throughout the years 2015 to 2018 and subsequent.

The class “loss of cover”, where previously sealed surfaces are re-opened, is also included in the IMCC layers, however from 2006 to 2015 this class is empty for Germany (data not shown). Merely in the IMCC layer 2015–2018, this class has an amount of 2700 ha which is negligible compared to 71,300 ha of newly sealed soils. In contrast to soil sealing, the term soil unsealing comprises the removal of artificial surfaces and a conversion of the area into a new, largely undeveloped area. The removal of sealing can provide restored soil for agriculture and forestry, in bare soil for pioneer species, a green area or a park (Tobias et al., 2018). Of course, soil functionality and quality might not be restored if the soil was once sealed, and the provision of ecosystem services is dependent on the soil conditions at the respective sites. Although sealed soils can uphold some of their functions, for example, supporting tree growth or water infiltration (Burghardt, 2006), it is still an impermeable surface that consumes the soil for the time it is sealed. For example, a study on soil unsealing in urban areas in Switzerland found that brownfield sites (derelict and underused industrial, commercial, or military sites, often with real or perceived soil contamination) have the potential to supply ecosystem services once the soils are unsealed and restored (Tobias et al., 2018).

## Conclusions

The study highlights the added value of a spatially explicit and quantitative assessment of soil losses due to sealing using geodatasets and remote sensing data. The use of the soil map BÜK 200 and selected evaluation schemes allowed the assessment of erosion resistance, runoff regulation, filter functions and yield potentials of soils in Brandenburg and Lower Saxony. The IMCC layers, despite their discrepancies with official soil loss statistics, are currently the only freely available option for spatially recording soil loss and approximating soil quality under sealing. It should be noted that this is a nationwide approach which is limited in scale, as the BÜK 200 soil map has a much lower spatial resolution than the IMCC data, but higher resolution soil maps could of course be used for more detailed regional analyses. In future steps, a site-specific assessment is necessary and will be included in order to ensure fairness and differentiation between different regions. The IMCC data are constantly being developed and improvements in data quality are expected in the coming years, so that remote sensing will increasingly support official statistics with explicit information on soil sealing. Furthermore, the introduced approach can be of great value to identify areas where soil loss has occurred and to assess the type of soil used in terms of its functions and potentials. This information can assist in the process of unsealing valuable areas, allowing stakeholders and decision-makers to re-evaluate previously sealed areas and reconsider future land use initiatives.

Ultimately, the use of high-resolution data can play a crucial role in supporting the goals outlined in the German National Strategy for Sustainable Development, which at the same time supports Germany’s commitments made to the UN (SDGs) and the EU (Soil Strategy). Monitoring soil, detecting soil loss due to soil sealing and making informed decisions based on this information, prioritizing the sustainable management and protection of soil resources as promoted by Good Agricultural Practice, will contribute to halting current levels of soil degradation and restoring soils to good health.

## Figures and Tables

**Fig. 9:**
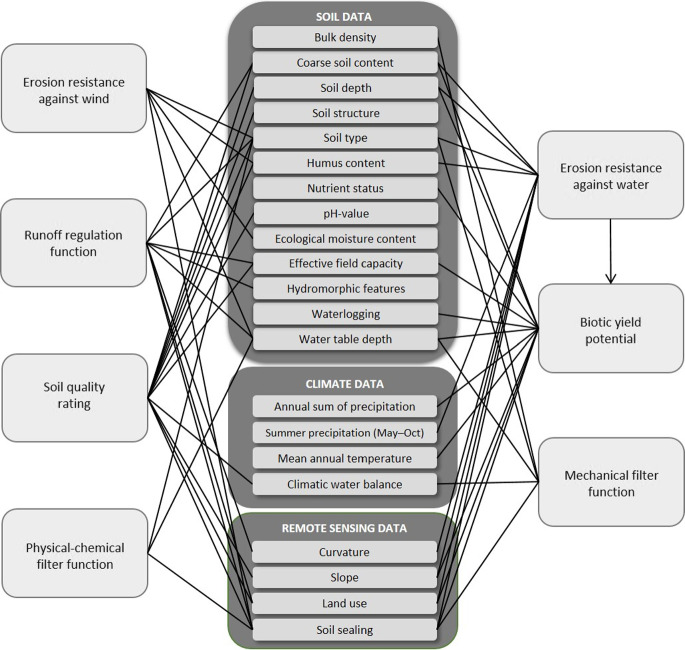
Schematic illustration of the seven soil functions and potentials (Marks et al., 1992; Müller et al., 2007) and their associated input variables for the calculations. The variables are divided thematically into soil data, climate data and remote sensing data

## Data Availability

Data is available on request from Annelie Säurich or Heike Gerighausen.
